# Advancing data management in local energy trading: European policy, state-of-the-art, and real-world practice in FEDECOM and STUNNED

**DOI:** 10.12688/openreseurope.21638.1

**Published:** 2025-11-03

**Authors:** Zia Lennard, Fayaz Ahmed, Matteo Porta, Katarina Stankovic, Carmina Bocanegra Yáñez

**Affiliations:** 1 R2M Solution SAS, Roquefort-Les-Pins, France; 2 R2M Solution Srl, Pavia, Italy; 3 RINA Consulting S.p.A, Genova, Italy; 4 Institut Mihajlo Pupin, Beograd, Serbia; 5 Elliot Cloud SL, Logrono, Spain

**Keywords:** data management, data governance, peer-to-peer energy trading, digitalisation, energy communities, interoperability, blockchain, smart grids, flexibility markets

## Abstract

Effective data management and digitalisation are central to the success of energy trading and exchange systems across Europe, with energy communities and distributed assets forming a new pillar of EU energy policy. This open letter critically reviews best practices in data management for local energy trading, beginning with the policy and technical context and a consolidated literature review, and then using the recently launched STUNNED and ongoing FEDECOM projects as in-depth case studies. Both projects highlight how robust, interoperable, and secure data infrastructures support the evolution from centralised utilities to decentralised, flexible, and consumer-centric energy markets. We analyse key architectural approaches, demonstrators, and integrated solutions, and conclude with recommendations for accelerating the adoption of these practices and facilitating collaboration across the European research and innovation landscape. This analysis reveals that aligning technical best practices with policy objectives not only enables operational efficiency, transparency, and consuler protection, but also accelerates the uptake of flexible, market-integrated energy communities across the EU.

## Introduction

The European Union’s drive for a clean, decentralised, and flexible energy system has placed energy communities and digitalisation at the heart of energy policy. The Clean Energy for All Europeans Package, together with the recast Electricity Market Directive (2019/944), establishes the framework for consumer empowerment, local energy communities, and flexible demand-side participation. These policies require robust digital infrastructures that can support the secure, real-time exchange of large volumes of diverse data—a challenge that is central to the modernisation of energy markets. Digitalisation—through advanced data management, open communication architectures, and robust cybersecurity—is widely recognised as an enabler of these ambitions. In parallel, the rapid evolution of market structures, including the rise of local and peer-to-peer (P2P) trading and flexibility services, is redefining the requirements for data management. The need to process granular, high-velocity data streams from a multiplicity of assets, market actors, and devices is driving the adoption of new architectures and standards. In this context, data management refers to the comprehensive processes of collecting, validating, storing, sharing, and analysing information from energy assets and market interactions, all within a framework that ensures security and regulatory compliance. The literature points to several interlinked best practices: comprehensive and secure data collection at the source, interoperable data models and APIs, advanced analytics for real-time operations, and open data governance models that balance transparency, privacy, and regulatory compliance.

At the intersection of policy ambition and technical innovation stand large-scale demonstrators, such as FEDECOM and STUNNED. These projects show how digital infrastructures—built on a foundation of state-of-the-art data management—are operationalising the new market and community paradigms envisaged by EU policy. The remainder of this paper draws together current best practice and concrete project experience to inform both ongoing research and practical deployment across Europe. FEDECOM and STUNNED serve as leading European testbeds for the practical implementation of such digital infrastructures, each addressing complementary aspects of interoperable, market-oriented data management. The following sections review the historical and technical context, analyse the state of the art, and present in-depth case studies of both projects, culminating in a comparative analysis and recommendations for future research.

## Literature review

### Historical context and evolution

The transformation of energy markets—from centralised systems with minimal data requirements focused mainly on billing and grid operations to decentralised, competitive environments—has greatly increased the volume, complexity, and strategic importance of data management ( Soto *et al.*, 2021; Zhou *et al.*, 2020). In earlier regimes, data was primarily used for billing and simple grid operations.

The liberalisation of energy markets in the late 20th century introduced new market actors, competitive trading, and dynamic pricing—each driving the need for more advanced data handling capabilities for pricing, risk management, and real-time market monitoring ( Zahraoui *et al.*, 2023). The subsequent adoption of the Harmonised Electricity Market Role Model (HEMRM) in Europe was a pivotal step toward standardising data exchange frameworks across borders ( Marinšek *et al.*, 2024). Meanwhile, the integration of renewable energy sources such as solar and wind, characterised by their intermittent and distributed output, has further amplified the demand for timely, accurate, and comprehensive data management ( Cuenca *et al.*, 2021).

More recently, the emergence of P2P trading, blockchain, smart contracts, and IoT technologies directly addresses these intensified data management challenges, enabling secure, decentralised, and transparent energy transactions among a wider array of market participants ( Bassey *et al.*, 2024). Collectively, these structural shifts have elevated data management from a supporting back-office function to a strategic capability that underpins market participation, operational reliability, and the integration of innovative business models in the energy sector. This evolving context sets the stage for a deeper analysis of the specific aspects and requirements of data management in modern energy systems, as explored in the following section.

### Key aspects of data management

The transition to smart grids and digital energy systems has fundamentally expanded both the complexity and the centrality of data management in energy trading and community operations.

Modern energy systems rely on the continuous collection of data from diverse sources—including smart meters, IoT sensors, and digital market platforms—which generate exponentially larger data volumes than conventional infrastructure. For example, smart meters capable of transmitting readings at 15-minute intervals now produce millions of data points per day across a single utility’s network. Each transaction in energy trading may reference dozens or even hundreds of supporting data points, including contractual, locational, and real-time consumption data. This explosion in data volume and diversity presents unprecedented challenges for data management, as traditional approaches struggle to aggregate, validate, and utilise such complex datasets for operational decision-making and market transparency.

Traditional data storage solutions, including legacy Energy Trading and Risk Management (ETRM) systems that rely on denormalisation and batch processing, have proven inadequate in the face of today’s transaction volumes and complexity, often leading to system slowdowns and limited scalability. As a result, many energy organisations are shifting to centralised data stores or data lakes, which offer scalable, secure, and reliable repositories for aggregating, normalising, and analysing critical market and operational data ( Capco, 2022). Newer decentralised approaches, such as Data Spaces, are also being developed to automate processes such as supplier switching and to enable secure, compliant data sharing across organisational boundaries ( Rülicke *et al.*, 2024).

High data quality is essential for effective energy exchange. Accurate meter readings are non-negotiable for correct billing and operational efficiency, while data completeness ensures that forecasting and market planning remain robust and uninterrupted. Consistency across data sources prevents discrepancies that could undermine regulatory reporting or grid reliability, and timeliness ensures that critical functions like real-time trading and outage management can be performed without delay ( Evangelista Bem *et al.*, 2023; Jha *et al.*, 2023). To support these aims, comprehensive data governance frameworks—encompassing standardisation, validation, security, and regulatory compliance—are increasingly seen as foundational requirements in energy data management ( Depiver, 2022).

Building on these foundations, energy organisations are increasingly leveraging advanced analytics to extract actionable insights, support grid optimisation, and develop sophisticated trading strategies ( Sun *et al.*, 2024). Visualisation tools are also crucial, enabling stakeholders to interpret complex data in accessible formats and thereby improve decision-making ( Brodny & Tutak, 2023). With the growing emphasis on consumer data protection ( Chiarini & Compagnucci, 2022), emerging solutions such as blockchain now offer secure, transparent methods for data sharing and transaction verification, especially in P2P markets where integrity and privacy are paramount ( Wang *et al.*, 2021). However, the adoption of these technologies also requires overcoming significant regulatory and integration challenges. As the complexity and criticality of data management in energy trading increases, the role of policy and regulation becomes ever more central—a theme addressed in the next section.

### European policy and regulatory framework

European energy policy not only shapes market structures but directly defines the technical requirements for data collection, exchange, and security in energy trading and community systems. A range of EU directives—including the Energy Efficiency Directive, the Renewable Energy Directive, and the EU Emissions Trading System (ETS)—establish binding requirements for the collection, monitoring, and reporting of energy data ( Bel & Joseph, 2018; Herweg, 2017; Hu *et al.*, 2024; Stoicescu *et al.*, 2023). For instance, the Energy Efficiency Directive mandates robust systems for tracking energy consumption improvements, while the Renewable Energy Directive requires accurate measurement and integration of renewable generation into national and EU-wide reporting systems. The ETS, as a market-based mechanism to reduce greenhouse gas emissions, relies fundamentally on the integrity of emissions data for both compliance and trading.

However, significant fragmentation persists across Member States, with differing regulatory structures resulting in disparities in energy prices and creating barriers to seamless, cross-border data exchange ( Faure-Schuyer *et al.*, 2017). Recognising these challenges, the EU has launched initiatives such as the Data Governance Act and is working towards a Common European Energy Data Space, both designed to facilitate secure, standardised, and interoperable data flows at scale. Achieving effective policy coordination and technical harmonisation remains essential for the realisation of a unified European energy market ( Teixeira *et al.*, 2014). These policy frameworks, while ambitious, create both challenges and opportunities for technical innovation in data management—a dynamic explored in the next section.

### Technological state-of-the-art

The rapid evolution of data management technologies is fundamentally reshaping how energy systems operate, addressing emerging challenges in data volume, velocity, and complexity. Modern smart grids rely on extensive networks of IoT devices that generate continuous, high-frequency data from across the system, enabling advanced real-time monitoring and automated control. At the core of these capabilities are multi-layered communication infrastructures—including Home Area Networks (HANs), Business Area Networks (BANs), and Neighbourhood Area Networks (NANs)—that must deliver high bandwidth, low-latency, and secure data transmission through a mix of wired and wireless technologies. Particularly, Advanced Metering Infrastructure (AMI) builds on these communication layers and represents a crucial part of the smart grid that turns conventional one-way metering into a two-way interactive network, enabling end-users and utilities to manage energy more intelligently. It can facilitate automated billing reads, remote meter monitoring, and control commands including dynamic price signals and remote service connection ( E-CIGRE, 2024). This comprehensive architecture ensures seamless data flow between end users, system operators, and market platforms. Emerging grid-sensing technologies, such as synchrophasors, provide operators with precise, real-time measurements of voltage and current phase angles, enhancing situational awareness and supporting grid stability even as the system becomes more dynamic and decentralised ( Jha *et al.*, 2023) ^9^.

Blockchain and distributed ledger technologies are increasingly employed to ensure data integrity, transparency, and security in energy trading. By automating and validating transactions via smart contracts, these systems can enable more efficient and reliable market operations ( Chiarini & Compagnucci, 2022; Evens *et al.*, 2023). In parallel, P2P trading models are redefining the energy landscape by allowing consumers and prosumers to buy and sell surplus energy directly, circumventing traditional utilities and leveraging decentralised, blockchain-based platforms. These innovations offer significant potential for market transparency and user empowerment but also introduce new challenges in integrating with existing regulatory and technical infrastructure ( WINSSolutions, 2023). However, integrating blockchain and decentralised trading with existing market infrastructures remains a significant technical and regulatory challenge.

Evidence from pilot projects suggests that blockchain-based platforms can substantially reduce trading costs—by as much as 30–40%—through automation, improved data transparency, and streamlined inter-system communications ( EnergiesMedia, 2023). These efficiency gains are motivating broader adoption, though practical implementation at scale remains hampered by interoperability and regulatory hurdles. Artificial intelligence (AI) and machine learning (ML) techniques are being deployed to forecast energy demand and generation with greater accuracy, optimise grid operations, and inform strategic trading decisions ( Bortoletto *et al.*, 2023). These tools enable more proactive, adaptive, and resilient system management. While these technological advances are rapidly expanding the capabilities of data management in energy systems, they also highlight the need for continuous attention to interoperability, standardisation, and cybersecurity as core pillars of sustainable digital energy infrastructure.

## Case studies

To illustrate how the best practices and technical challenges discussed in the previous sections are being addressed in real-world settings, this section presents two major European projects—STUNNED and FEDECOM. Both projects serve as leading testbeds for the practical implementation of advanced data management in energy communities, offering valuable insights into both the opportunities and remaining obstacles in the digitalisation of energy trading.

### The STUNNED project

The STUNNED project (Horizon Europe Grant 101172968), implemented by a consortium of research institutions, technology providers, and market actors, exemplifies a cutting-edge approach to operationalising advanced data management in European energy communities. The project aims to unlock the flexibility and demand response potential of residential and industrial buildings aggregated in energy communities and energy districts by deploying secure, modular digital platforms capable of aggregating diverse energy assets and enabling innovative market participation models. Key objectives include the integration of building energy management systems (EMS), distributed energy resources (DERs), and electric vehicle (EV) charging infrastructure, alongside the development of advanced analytics for system optimisation and the validation of scalable, replicable business models for flexibility services.

Technically, STUNNED’s digital architecture is designed as a modular, interoperable platform stack to ensure secure, efficient, and privacy-compliant data flow between distributed assets, local EMS, and market interfaces. Leveraging IoT and industrial-grade control and automation, STUNNED aims to enable integration at different application layers through both industry-standard and vendor-specific protocols. Standardised and secure communication protocols support both real-time and batch data exchanges, facilitating robust and flexible data management across varied operational contexts.

Modular design of the STUNNED platform ensures compatibility with arbitrary type of legacy equipment onsite, from industrial-grade EMS systems to lightweight IoT thermostats in buildings. It envisions local data-management hubs as gateways to distributed assets, allowing for decentralised data acquisition and real-time local control while safeguarding the operational processes of flexibility providers. To address interoperability challenges caused by diverse range of assets and external platforms, the design of STUNNED platform includes a dedicated semantic interoperability module, serving as a unified dictionary that comprehensively defines the relevant features required to accurately describe each asset and its interface.

STUNNED Cloud Platform (SCP) is the core of the STUNNED architecture, and manages the components that govern the whole infrastructure: Ontology, Semantics, Time Series, KPI definitions, Operational Hierarchies, and Access Control are part of the SCP.

The Broker service attached to the SCP is the bridge within the STUNNED Cloud Platform and external agents deployed on each location, to both send data and receive instructions.

Data is organised in hierarchies, and gathered within the STUNNED Cloud Platform Data Lake, from where it’s shared with authorised partners via its interoperability layer (API). That modular and interoperable design enables both local and remote analytics to support optimisation and effective participation in energy markets with a complete view of the data and its semantic meaning.

Advanced algorithms—including game-theoretic and multi-objective optimisation—form the backbone of the platform’s forecasting, bidding, and demand response capabilities. Compliance with FAIR (Findable, Accessible, Interoperable, Re-usable) data principles and the General Data Protection Regulation (GDPR) is embedded throughout, ensuring privacy and transparency in all operations.

The platform’s technical design prioritises modularity and interoperability, allowing for seamless integration of new devices and the rapid scaling of community participation. Security is upheld through end-to-end encryption, role-based access controls, and ongoing system monitoring for vulnerabilities. Notably, STUNNED’s architecture is innovative in its planned integration of flexibility services with both local grid operators and wholesale market platforms, allowing for scalable participation in multiple market layers and regulatory environments.

Demonstrators within STUNNED are being deployed across a variety of regulatory and market contexts, including residential districts, commercial properties, industrial sites, and mixed-use developments (Italy, France, and Spain). Each pilot site integrates a suite of distributed assets with the STUNNED platform, rigorously testing both the technical integration and the business model innovations in operational environments. By deploying a modular and interoperable digital platform, STUNNED aims to validate not only the technical feasibility but also the market viability of advanced data management strategies for energy communities. The project’s capacity to support automated data sharing, secure transactions, and real-time optimisation is expected to enhance grid flexibility, increase consumer and prosumer participation, and generate robust business cases for community-led trading and demand response. Precisely, the impact of STUNNED solution will be measured against a designated set of key performance indicators (KPIs) including peak power reduction, self-consumption, self-sufficiency, hours of flexibility activated for grid services, operating costs and revenues, among other. The successful demonstrators will provide actionable blueprints for replication in diverse European contexts and will inform ongoing policy development on data governance and market integration.

### The FEDECOM project

The FEDECOM project (Horizon Europe Grant 101075660), coordinated by a multidisciplinary consortium encompassing technology developers, utilities, research institutions, and community representatives from several EU countries, advances a federated “system-of-systems” approach to data management in energy exchange and trading. Moving beyond the limitations of isolated pilots, FEDECOM is designed to enable cross-border, multi-vector energy trading and flexibility sharing through interoperable digital platforms and robust, open data management architectures. The central objectives are to demonstrate the scalability and interoperability of digital platforms supporting diverse energy assets—including electricity, heating, mobility, and hydrogen—across communities, while maintaining open, secure, and GDPR-compliant data management and governance frameworks.

FEDECOM’s technical strategy is built upon several foundational pillars. First, secure communication middleware enables both real-time and batch data acquisition from a heterogeneous range of field assets and pilot sites, ensuring data can be ingested and utilised regardless of local technology configurations. Second, a canonical data model (CDM), mapped to international standards, harmonises data semantics and structures to facilitate interoperability among a wide variety of stakeholders, platforms, and market actors. Third, the project integrates the Grid Singularity Exchange (GSY DEX), a blockchain-based decentralised energy marketplace, to support transparent, secure, and automated P2P and cross-community trading. This system enables real-time performance monitoring, automated measurement and verification (M&V), and seamless settlement of transactions. The analytics engine further supports the tracking of key performance indicators such as community self-sufficiency, self-consumption, peak load management, and user engagement. Governance is maintained through a comprehensive data management plan fully aligned with FAIR and GDPR principles, guaranteeing data integrity, privacy, and regulatory compliance.

FEDECOM’s demonstrator activities are structured around three main “federations,” each representing a distinct technical and regulatory context. The Virtual Green Hydrogen Federation in Spain brings together municipal buildings, renewable generation, and hydrogen production in a unified trading and flexibility ecosystem, demonstrating secure data sharing and automated settlement across diverse actors. The Residential Hydropower Federation in Switzerland, coordinated by SUPSI and local partners, aggregates distributed hydropower, storage, and community loads, optimising local use and market participation through interoperable and secure data management. The E-Mobility and Local Energy Federation, spanning Belgium and the Netherlands, focuses on integrating commercial and community sites—including the Brussels Brico Retail Community, Voorhout Village, and Besix HQ & Eemnes Community—connecting EV infrastructure, photovoltaic (PV) generation, battery storage, and flexible loads under a common digital umbrella.

Through these live demonstrations, the FEDECOM platform is being actively deployed from field-level data acquisition to blockchain-based market operations, enabling intra- and inter-community optimisation, real-time energy and flexibility exchange, automated performance verification, and transparent remuneration. KPIs tracked include latency and reliability of data transactions, user privacy compliance, market integration efficiency, and levels of community and prosumer participation.

The impact of FEDECOM is anticipated to be substantial. By enabling secure, interoperable, and scalable energy trading among a diverse range of actors, FEDECOM increases prosumer and community participation in local and regional markets, enhances grid flexibility and resilience, and accelerates the adoption of renewable and multi-vector energy solutions. Data, insights, and lessons from these demonstrators are synthesised to inform policy, support market design, and create replicable blueprints for market-ready, consumer-centric energy systems across the European Union, thus advancing Europe’s clean energy transition and digitalisation objectives.

## Comparative analysis: synergies, best practices, and lessons learned

FEDECOM advances a system-of-systems architecture grounded in a canonical data model and a decentralised marketplace (GSY DEX) for transparent, secure, and automated P2P and inter-community transactions, whereas STUNNED emphasises modular, federated data management with secure, standardised communications and strong local control. Both initiatives converge on the foundational principle that interoperability—across data models, application programming interfaces (APIs), and market interfaces—is essential for achieving scalable, replicable, and market-ready solutions. FEDECOM illustrates a system-of-systems architecture grounded in a canonical data model and blockchain-based trading through the Grid Singularity Exchange (GSY DEX), enabling transparent, secure, and automated P2P and community-to-community transactions. In contrast, STUNNED adopts a modular, federated data management approach, prioritising secure, standardised communications and local control, which facilitates the flexible integration of heterogeneous assets and supports seamless aggregation of flexibility services at both the community and district levels.

Both projects demonstrate that secure and scalable communication infrastructures, coupled with advanced analytics engines, are fundamental for ensuring operational reliability, real-time responsiveness, and transparency in market operations. FEDECOM’s approach underscores the value of a decentralised marketplace, providing intrinsic transparency, auditability, and automated settlement, whereas STUNNED’s focus on federated, modular platforms highlights the need for adaptability across different regulatory and business environments. Importantly, both projects incorporate robust data governance frameworks—aligned with FAIR (Findable, Accessible, Interoperable, Re-usable) principles and the General Data Protection Regulation (GDPR)—demonstrating that privacy, compliance, and open governance are now prerequisites for regulatory approval and stakeholder trust. Both case studies also respond directly to challenges highlighted in the literature, including the need to process high-velocity and high-volume data streams, the importance of data quality and standardisation, and the operational complexities introduced by distributed resources and P2P trading models.

Despite their shared objectives, FEDECOM and STUNNED diverge in their primary architectural paradigms and implementation strategies. FEDECOM leverages blockchain technology to address issues of market trust, transparency, and automated settlement at scale, whereas STUNNED employs a federated approach to enhance local autonomy and facilitate the rapid adaptation to new asset types and evolving market requirements. Taken together, these complementary approaches point toward a harmonised European energy data ecosystem, where modularity, interoperability, and robust governance work synergistically to unlock the full value of flexible, consumer-centric energy communities. The insights from both projects not only validate best practices identified in the academic literature but also offer actionable blueprints for future projects and policy frameworks seeking to accelerate the clean energy transition in Europe through advanced data management.

## Conclusions and recommendations for future research

The collective experience of the STUNNED and FEDECOM projects demonstrates that aligning technical best practices with European policy objectives can unlock substantial new value in energy trading, flexibility markets, and community participation. Both projects show that achieving genuine interoperability, robust security, and open data governance is feasible with today’s digital technologies, though significant challenges remain regarding scalability, user engagement, cross-border harmonisation, and regulatory adaptation.

For practitioners and researchers, several concrete recommendations emerge from these case studies. First, future data management initiatives should prioritise modular, standardised architectures that enable seamless integration and interoperability across platforms, assets, and regulatory regimes. Second, security measures—including end-to-end encryption, strong authentication, and privacy-by-design principles—should be integrated at every layer of system design and operation, to comply with GDPR and FAIR requirements and to foster lasting trust among consumers and market participants. Third, frameworks for data quality—addressing accuracy, completeness, consistency, and timeliness—should be institutionalised, with continuous validation and monitoring to support both operational efficiency and regulatory compliance. Fourth, the development and deployment of analytics and decision-support tools should be centered on the needs of users, including communities, prosumers, and grid operators, to ensure that data-driven insights are both accessible and actionable. In parallel, deploying FAIR-aligned governance with clear role-based access controls and auditability is essential to maintain trust while scaling data sharing across organisational and national boundaries.

Further research is urgently needed in several areas: the development and standardisation of frameworks that facilitate the integration of advanced analytics and decentralised trading into legacy grid environments; the design of robust, user-friendly platforms that lower barriers to community participation and data governance; and the harmonisation of cross-border regulatory and market practices to enable frictionless, pan-European energy data exchange and trading. Evaluating consumer and stakeholder acceptance of these digital platforms across Europe is also essential, given the diversity of regulatory, technical, and cultural contexts. Finally, clustering and knowledge exchange—through joint workshops, demonstration projects, and thematic working groups—should be expanded to accelerate innovation, share lessons learned, and inform ongoing policy development at both national and European levels.

By addressing these priorities and harnessing technological advancements, the European energy sector can fully realise the potential of modern data management, creating sustainable, resilient, and participatory energy systems that support Europe’s ambitious decarbonisation and digitalisation goals.

## Disclaimer

The views expressed in this article are those of the authors. Publication in Open Research Europe does not imply endorsement by the European Commission.

## Ethics and consent

Ethical approval and consent were not required.

## Data Availability

No data are associated with this article.
