# Supplementary material for: Advancing data management in local energy trading: European policy, state-of-the-art, and real-world practice in FEDECOM and STUNNED
Source: Open Res Eur. 2026 Jan 14;5:334. Originally published 2025 Nov 3. [Version 2] doi: 10.12688/openreseurope.21638.2 (PMC13019032; doi:10.12688/openreseurope.21638.2)
Supplement: Peer review history

# Reviewer report: Reviewer response for version 2

**DOI:** 10.21956/openreseurope.24544.r70955

**Published:** 2026-04-14

**Authors:** Binod Koirala

**Affiliations:** 1 Federal Laboratories for Materials Science and Technology,, Überlandstrasse 129,, 8600 Dübendorf,, Switzerland

The open letter is a nice attempt. It highlights the data management practices employed in FEDECOM and STUNNED projects. 

 However, it is not well justified why the focus is on these projects. It is a mere coincidence, or are the data management practices in both projects actually complementary? It also lacks details on, for example, semantic ontologies, knowledge graphs, and digital twins used in these projects to ensure harmonization in data management practices. 

 The lesson learned and the recommendation for future research are well elaborated and understandable. It can be further extended to include data harmonization, data security, ethical considerations, and the role of novel AI tools.

Where applicable, are recommendations and next steps explained clearly for others to follow? (Please consider whether others in the research community would be able to implement guidelines or recommendations and/or constructively engage in the debate)

Partly

Does the article adequately reference differing views and opinions?

Yes

Are all factual statements correct, and are statements and arguments made adequately supported by citations?

Yes

Is the rationale for the Open Letter provided in sufficient detail? (Please consider whether existing challenges in the field are outlined clearly and whether the purpose of the letter is explained)

Yes

Is the Open Letter written in accessible language? (Please consider whether all subject-specific terms, concepts and abbreviations are explained)

Yes

Reviewer Expertise:

Energy communities, digital twins, energy system modelling, urban energy systems

I confirm that I have read this submission and believe that I have an appropriate level of expertise to confirm that it is of an acceptable scientific standard.

# Reviewer report: Reviewer response for version 2

**DOI:** 10.21956/openreseurope.24544.r71271

**Published:** 2026-03-25

**Authors:** Lefteris Topaloglou

**Affiliations:** 1 University of Western Macedonia, Kozani, Greece

**1. Summary of the Article**

 The open letter 'Promoting data management in local energy trading: European policy, recent developments and practical applications in the FEDECOM and STUNNED programmes', examines the role of data management in implementing modern, decentralised energy systems across Europe. The article contextualises the discussion within the evolving political landscape of the EU, emphasising the growing importance of digitisation, interoperability, and governance within energy communities.

 The article takes a practical approach, combining:

 - a review of policy and literature on energy data management;

 - a discussion of technological developments (e.g. smart grids, the Internet of Things (IoT) and blockchain);

 - Examples from two projects in the 'Horizon Europe' programme: STUNNED and FEDECOM.

 Its main contribution is to connect political objectives, theoretical ideas and real-world applications, while also making recommendations to improve the interoperability, scalability and governance of data-driven energy trading systems.

**2. Overall Assessment**

 The Open Letter successfully addresses a relevant and timely issue, combining political, technical and practical perspectives. It is well structured and informative, and the use of real projects adds value.

 However, while the document is strong in conceptual terms, several aspects require improvement to achieve full scientific credibility.

 - The justification and definition of the problem are not always clear.

 - The engagement with different perspectives is limited.

 - Some claims are not adequately supported by references.

 - The recommendations, while useful, remain somewhat general.

**3. Detailed Evaluation**

**3.1 Rationale and Problem Framing**

 Justification and definition of the problem

**Advantages**: (1) The article clearly identifies the key challenges (e.g. interoperability, data governance and fragmentation). (2) It sets out a general objective of connecting policy, literature and application.

**Limitations:** (1) The main problem is not clearly defined from the outset. (2) The justification is somewhat vague and descriptive, particularly in the introduction and literature review. (3) Although a guiding question exists, it is not emphasised enough.

**Recommendations for improvement:** Clearly state the main problem in the first 1–2 paragraphs. (1) What is the main problem? (2) Why is it important now? (3) What gap does this open letter cover?

 Also summarise the justification for the Open Letter in 2–3 sentences. Additionally, state explicitly: This Open Letter addresses the gap between X and Y by [...]

**3.2 Referencing Differing Views and Opinions**

**Advantages:** (1) The article refers to a wide range of literature. (2) It covers multiple fields (politics, technology and markets).

**Limitations:** (1) It does not explicitly compare or contrast different perspectives. (2) There is little discussion of: - Competitive approaches (e.g. centralised versus decentralised systems);

 - Contradictory evidence;

 - Exchange or debate.

**Recommendations:** (1) Include brief comparative discussions, for example: Blockchain versus traditional platforms; centralised versus federated data architectures; and political versus market-driven approaches. (2) Add proposals such as: 'While some studies support X, others highlight Y...' (3) Provide a brief discussion of the limitations or criticisms of current approaches.

**3.3 Accuracy and Use of Citations**

**Advantages**: (1) The article is generally well referenced. (2) Many claims are supported by references.

**Limitations**: Some statements are overly general (e.g. 'widely recognised', 'significant impact') and not directly supported by references. (2) The descriptions of the works rely heavily on storytelling rather than evidence.

**Recommendations:** (1) Add references to:

 - General claims about trends or consequences

 - Quantitative statements

 - Claims about the effectiveness of technologies

 (2) Where it is not possible to provide references, limit the statements to 'expected to' or 'early indications suggest'. Where it is not possible to provide references, limit the statements to phrases such as 'expected to' or 'early indications suggest'. (3) Clarify whether statements in work sections are empirical findings or expectations.

**3.4 Recommendations and Next Steps**

**Advantages:** The recommendations are clearly structured and relevant. They align with the key challenges that have been identified.

**Limitations:** (1) The recommendations are often high-level (strategic rather than operational) and do not always clearly connect to specific findings or examples. (2) There are limited guidelines on how to implement them.

**Recommendations:** (1) Make the recommendations more applicable by adding examples (e.g. from FEDECOM/STUNNED), specifying who should act (e.g. researchers, policymakers or industry) and indicating how (e.g. tools, frameworks or steps). (2) Strengthen the connection by starting recommendations with 'Based on the comparison of STUNNED and FEDECOM, we recommend...'.

**4. Key Required Revisions (Must Address)**

 To ensure that the article is scientifically sound, the following must be addressed:

**Essential:** (1) Strengthen the formulation of the problem and justification. The main problem and contribution must be formulated clearly, concisely and in a timely manner. (2) Improve the referencing. Ensure that all key claims are supported by references or are appropriately substantiated.

**Recommended:** (1) Increase the analytical depth. Make more explicit reference to different perspectives. (2) Facilitate the implementation of recommendations. Clearly link them to the findings and provide implementation guidelines.

**5. Final Recommendation**

 The Open Letter is valuable and relevant, and is almost ready for indexing. However, it requires moderate revisions to enhance conceptual clarity, analytical depth, and substantiation. With these improvements, it can make a significant contribution to the field of energy transition and digital energy systems.

Where applicable, are recommendations and next steps explained clearly for others to follow? (Please consider whether others in the research community would be able to implement guidelines or recommendations and/or constructively engage in the debate)

Partly

Does the article adequately reference differing views and opinions?

Partly

Are all factual statements correct, and are statements and arguments made adequately supported by citations?

Partly

Is the rationale for the Open Letter provided in sufficient detail? (Please consider whether existing challenges in the field are outlined clearly and whether the purpose of the letter is explained)

Partly

Is the Open Letter written in accessible language? (Please consider whether all subject-specific terms, concepts and abbreviations are explained)

Yes

Reviewer Expertise:

Just Energy Transition, Energy, Climate & Environmental Justice, Place-Based Governance

I confirm that I have read this submission and believe that I have an appropriate level of expertise to confirm that it is of an acceptable scientific standard, however I have significant reservations, as outlined above.

# Reviewer report: Reviewer response for version 1

**DOI:** 10.21956/openreseurope.23407.r63563

**Published:** 2025-12-24

**Authors:** Loïc Cobut

**Affiliations:** 1 UCLouvain Saint Louis Bruxelles, Bruxelles, Belgium

Overall speaking, this is an interesting piece on a timely topic. The two projects are both insightful, and the authors bring interesting thoughts forward. I would encourage the authors to consider my comments, which could help improve the piece:

  

- **Abstract:** it is an heavy abstract which could be more concise and to the point, especially if we are talking about an open letter. It is a bit unclear what is meant by critical review of best practices (through what lenses?), “policy and technical context”, which are too broad concepts, and then linking with ongoing projects (why?) - all that in one sentence. What you qualify as the European research and innovation landscape is broad and abstract, please identify your audience more precisely. In the abstract you talk about an analysis, but then I understand that this is an open letter – you need to clarify what you do with this piece.

 

- **Introduction**
-
  - Local energy communities do not exist under EU law, do you mean renewable energy communities or citizen energy communities, or both?
  - Adding a few references in the first paragraph would help embed your open letter in existing literature, so you situate it directly and it helps the reader to understand where you speak from.
  - This introduction lacks a clear problematisation, even it we talk about an open letter and not a research article: what is the problem at stake? What are the objectives of this piece? What is the leading question (and hypothesis)? In what literature does this article finds itself?
- **Literature review**
  - I’m not sure to understand the need of a literature review in an open letter? It can be done, but then you need to justify and explain why you do this.
  - The literature review is wide and opens many doors. However, it does not precise the type of problem that is at the center of this paper. The authors should have in mind that the piece should address a concise problem, shed light on a phenomenon that is puzzling for some reasons. Here, the literature review rather describes history, policy and technology evolution.
- **Case studies**
  - This section operates both as a method section (presenting cases of what method?) and a presentation of the empirical framework of the research (two EU projects). However, the main issue is that there is no proper method that is presented. In the next section, a comparative analysis is being led without link to any research method. As a next step, I suggest that authors present their comparative method at the beginning of this section, in order to provide with a clearer and more structured research framework. But then again, is this what the authors want to do in an open letter (having case studies and a comparative analysis)?
  - When presenting STUNNED, the 7-8 first paragrahps offer descriptive content about this project but as there is no clear research objective, it’s hard for the reader to understand why this is helpful information. Authors need to guide readers a bit more.
  - Within STUNNED, you might want to be a bit more specific : where exactly? Which energy communities are involved and how?
  - I understand that you don’t want to publish a research article where you present your results but at the same time, you might want to present preliminary results from STUNNED and FEDECOM to tease the readers.
- **Comparative analysis**
  - You first need to explain why you compare those two projects? Why those two and not other projects? Why 2 and not 3 or 4 or 7? How did you select those 2 projects specifically? Again, having clear objectives for this piece would help you to justifiy all these choices.
  - Links with existing literature are almost non-existent, it can not be absent and at the same time an argument that is used to value your demonstration (at the end of this analysis).
- **Conclusion**
  - You start by referring to a demonstration – which is the central issue of this piece: I miss a clear demonstration in the analysis. I miss an analysis that brings forward a sery of arguments that help demonstrate that an initial hypothesis is verified or not.
  - You have recommendations for practitioners and researchers: Are these recommendations only for them or do you also want to inform policy makers (this is not clear throughout the paper).
  - There needs to be clearer articulations between your recommendations and your comparative analysis.

Where applicable, are recommendations and next steps explained clearly for others to follow? (Please consider whether others in the research community would be able to implement guidelines or recommendations and/or constructively engage in the debate)

Partly

Does the article adequately reference differing views and opinions?

Partly

Are all factual statements correct, and are statements and arguments made adequately supported by citations?

Partly

Is the rationale for the Open Letter provided in sufficient detail? (Please consider whether existing challenges in the field are outlined clearly and whether the purpose of the letter is explained)

Partly

Is the Open Letter written in accessible language? (Please consider whether all subject-specific terms, concepts and abbreviations are explained)

Yes

Reviewer Expertise:

Energy communities, energy governance, European politics, transition research.

I confirm that I have read this submission and believe that I have an appropriate level of expertise to confirm that it is of an acceptable scientific standard, however I have significant reservations, as outlined above.

# Response

**Published:** 2026-01-06

**Authors:** Zia Lennard

**Affiliations:** R2M Solution, France

We sincerely thank the reviewer for their careful reading of the manuscript and for their thoughtful and constructive comments. We appreciate the recognition of the relevance and timeliness of the topic, as well as the acknowledgement of the value of the FEDECOM and STUNNED project experiences. The reviewer’s comments have helped us significantly improve the clarity, framing, and coherence of the Open Letter. Our responses and corresponding revisions are summarised below.

**Abstract**

 In response to the reviewer’s concern that the abstract was overly dense and insufficiently clear for an Open Letter, we have rewritten the abstract in full. The revised abstract is more concise and explicitly clarifies the nature of the contribution, namely that the paper is a practice-oriented Open Letter rather than a formal empirical analysis. We now clearly identify the intended audience (researchers, technology providers, project developers, and policymakers) and explain that FEDECOM and STUNNED are used as illustrative examples to bridge European policy objectives, selected literature, and real-world implementation. **Introduction and problem framing**

 We have revised the Introduction to improve conceptual clarity and problematisation. First, we clarified the legal terminology around energy communities, explicitly referring to Renewable Energy Communities and Citizen Energy Communities as defined under EU law, and explaining how the term “energy communities” is used throughout the paper for readability. Second, we strengthened the embedding of the Open Letter within existing debates by adding explicit problem framing. A new paragraph now clearly identifies data management as a critical bottleneck for scalable and interoperable energy trading, despite increasing policy attention and technological progress. This paragraph also introduces a guiding question that frames the purpose of the Open Letter, namely how contemporary European research projects operationalise best practices in data management and what lessons can be drawn for future research, innovation, and policy design.

**Role and scope of the literature review**

 In response to the reviewer’s question regarding the appropriateness of a literature review in an Open Letter, we have added explicit justification at the beginning of the literature section. We clarify that the literature review is not intended to be exhaustive, but rather serves to contextualise recurring policy-driven and technical challenges in data management for energy exchange and trading. We further strengthened the link between the literature review and the rest of the paper by explicitly stating that it provides the analytical backdrop against which the two projects are discussed.

**Case studies and comparative logic**

 To address concerns regarding the absence of a formal method and the rationale for selecting FEDECOM and STUNNED, we have clarified the comparative logic at the beginning of the case study section. We explicitly state that the projects are not presented as case studies in the methodological sense, but as illustrative examples drawn from the authors’ direct involvement in European research and innovation projects. We explain why these two projects were selected, namely because they address similar data management challenges under common European policy constraints, while differing in scope, maturity, and architectural approach.

 Within the STUNNED section, we added guiding sentences to explain how the technical description relates to the data management challenges identified in the literature, and we clarified the rationale for the selection of demonstration contexts. These additions aim to guide the reader and make the descriptive content more analytically meaningful, without overstating preliminary results.

**Comparative analysis and links to literature**

 We have strengthened the framing of the comparative analysis by explicitly explaining why STUNNED and FEDECOM are compared and what analytical value this comparison provides. In addition, we reinforced the link between the comparative insights and existing literature by explicitly noting how the observed challenges and best practices resonate with findings reported in prior work on interoperability, data governance, and platform-based energy systems.

**Conclusions, audience, and recommendations**

 In the Conclusions section, we clarified the target audience of the recommendations by explicitly stating that they are intended not only for researchers and technology providers, but also for policymakers involved in shaping regulatory and governance frameworks for digital energy markets. We also strengthened the articulation between the comparative analysis and the recommendations by clearly stating that the recommendations are directly derived from the comparative insights discussed earlier in the paper. Finally, we adjusted the language to avoid any ambiguity around “demonstration,” emphasising that the projects illustrate and exemplify best practices rather than formally proving hypotheses. Overall, we believe these revisions substantially improve the clarity, coherence, and positioning of the Open Letter, while remaining fully aligned with its intended format and purpose. We are grateful to the reviewer for helping us sharpen the contribution and better communicate its scope and value to the research, innovation, and policy communities.

# Reviewer report: Reviewer response for version 1

**DOI:** 10.21956/openreseurope.23407.r63562

**Published:** 2025-11-25

**Authors:** Olawale M Popoola

**Affiliations:** 1 Tshwane University of Technology, Pretoria, Gauteng, South Africa

**Open Letter**

 Title: Advancing data management in local energy trading: European policy, state-of-the-art, and real-world practice in FEDECOM and STUNNED

**Article Type**: Open Letter

 The Open Letter presents the role of data management in modern energy systems, focusing on policy and technical aspects, with the STUNNED and FEDECOM projects as case studies. The authors traced the challenges of data management in modern energy systems to the explosion in data volume and diversity. However, it could be improved with more details. Please see below my comments.

**Introduction:**

 -Introduced API before abbreviating for the sake of the reader(s) who may not understand the abbreviation at a glance.

 -Authors should introduce the Motivation/contribution/novelty of the work before reporting the organization of the article.

**Case Studies**

 Under the FEDECOM, the performance metrics (KPIs) for achieving the central objectives in demonstrating the scalability and interoperability of digital platforms supporting diverse energy assets were listed to be latency and reliability of data transactions, user privacy compliance, market integration efficiency, and levels of community and prosumer participation. However, the corresponding KPIs for achieving the key objectives of on STUNNED project (which include the integration of building EMS, DERs, and EV charging infrastructure, alongside the development of advanced analytics for system optimisation and the validation of scalable, replicable business models for flexibility services) were not captured.

Where applicable, are recommendations and next steps explained clearly for others to follow? (Please consider whether others in the research community would be able to implement guidelines or recommendations and/or constructively engage in the debate)

Partly

Does the article adequately reference differing views and opinions?

Yes

Are all factual statements correct, and are statements and arguments made adequately supported by citations?

Partly

Is the rationale for the Open Letter provided in sufficient detail? (Please consider whether existing challenges in the field are outlined clearly and whether the purpose of the letter is explained)

Yes

Is the Open Letter written in accessible language? (Please consider whether all subject-specific terms, concepts and abbreviations are explained)

Yes

Reviewer Expertise:

Energy and Power systems

I confirm that I have read this submission and believe that I have an appropriate level of expertise to confirm that it is of an acceptable scientific standard.

# Response

**Published:** 2026-01-06

**Authors:** Zia Lennard

**Affiliations:** R2M Solution, France

We sincerely thank the reviewer for their careful reading of the manuscript and for their constructive and supportive comments. We are grateful for the positive assessment of the relevance, accessibility, and overall contribution of the Open Letter. In response to the specific suggestions raised, we have made the following revisions:

1. **Clarification of abbreviations in the Introduction**
  We have revised the Introduction to ensure that technical abbreviations are introduced in full at first occurrence. In particular, “application programming interface (API)” is now explicitly defined before the abbreviation is used, improving accessibility for readers from diverse disciplinary backgrounds.
2. **Clear articulation of motivation, contribution, and novelty**
  To strengthen the framing of the paper, we have added a dedicated paragraph at the end of the Introduction, immediately before the description of the article structure. This new paragraph explicitly sets out the motivation for the Open Letter, clarifies its main contributions, and highlights its novelty in linking European policy, state-of-the-art research, and real-world implementation through the STUNNED and FEDECOM projects.
3. **Explicit KPIs for the STUNNED case study**
  In Section 3.1, we have added a clear and explicit description of the key performance indicators used to assess progress in the STUNNED project. These KPIs are directly aligned with the project’s core objectives, including flexibility activation, forecasting and optimisation performance, data exchange reliability and latency, participation of buildings and prosumers, and the validation of flexibility-related business models. This ensures symmetry with the KPI treatment already provided for FEDECOM and strengthens the comparative value of the case studies.
4. **Improved clarity and actionability of recommendations**
  In the Conclusions and Recommendations section, we have added a clarifying sentence explicitly stating that the proposed recommendations are intended to be directly actionable by future European research projects. This reinforces the practical relevance of the guidance provided and clarifies how the research community can build upon the insights presented.

We appreciate the reviewer’s confirmation that the rationale, referencing of perspectives, and overall accessibility of the Open Letter are sound. The reviewer’s suggestions have helped us improve clarity, balance, and completeness, and we thank them again for their valuable input.
